# Solar Drying of Mangoes: Opportunities for Combating Vitamin A Deficiency in Sub-Saharan Africa

**DOI:** 10.3390/foods14223979

**Published:** 2025-11-20

**Authors:** Paula Viola Salvador, Federico Gómez Galindo

**Affiliations:** 1 Division of Food and Pharma, Department of Process and Life Science Engineering, Lund University, 221 00 Lund, Sweden; paula.viola@ple.lth.se; 2 Centre of Excellence in Agri-Food Systems and Nutrition, Faculty of Agronomy and Forestry Engineering, Eduardo Mondlane University, Maputo 257, Mozambique

**Keywords:** vitamin A deficiency (VAD), solar drying, mango preservation, β-carotene retention, food-based interventions

## Abstract

Vitamin A deficiency (VAD) remains a severe health issue in sub-Saharan Africa, causing blindness, illness, and child mortality. In Mozambique, about 69% of children under five are affected, highlighting the short-term impact and donor dependence of supplementation programs. Mangoes (*Mangifera indica* L.), rich in provitamin A carotenoids, offer a sustainable, food-based strategy to reduce VAD, but their high perishability and postharvest losses of 20–40% limit their impact. This review combined analysis of 21 studies on solar drying of mangoes in Africa with interviews from health directors in three districts of Inhambane Province, Mozambique, to assess both technical and practical aspects of mango utilization. Findings show that improved solar dryers reduce drying time by up to 40 h compared with open-sun drying, achieve safe moisture content below 12%, and retain 60–90% of β-carotene—significantly higher than the 40–55% typical of open-sun methods. One hundred grams of solar-dried mango can meet 60–100% of a child’s or 50–70% of a woman’s daily vitamin A needs. Despite these advantages, interviews revealed limited community adoption and persistent dependence on supplementation. To bridge this gap, initiatives must enhance training, access to affordable dryers, and policy integration to turn seasonal mango surpluses into sustainable, year-round nutrition solutions.

## 1. Introduction

Vitamin A deficiency (VAD) represents a significant public health issue in many low and middle-income countries, particularly in sub-Saharan Africa. It is a leading cause of preventable childhood blindness and is associated with impaired immunity and an increased risk of mortality from infections such as diarrhea and measles [1].

Although the global prevalence of VAD was estimated at 39% in 1991 and had declined to 29% by 2013 [2], more recent analyses highlighted that the burden remains disproportionately high in sub-Saharan Africa, where prevalence has been reported at around 48% [3]. In 2013 alone, VAD was associated with approximately 94,500 diarrhea-related deaths and 11,200 measles-related deaths in children under five, with the vast majority occurring in sub-Saharan Africa and South Asia [2,4]. Country-level estimates further underlined the severity of the problem: in Mozambique, 69% of children aged 6–59 months were reported to have suffered from VAD in 2015, compared to 11% of mothers in 2019 [5,6]. Together, these findings point to the persistent and urgent need for sustainable food-based interventions.

**Figure 1:**
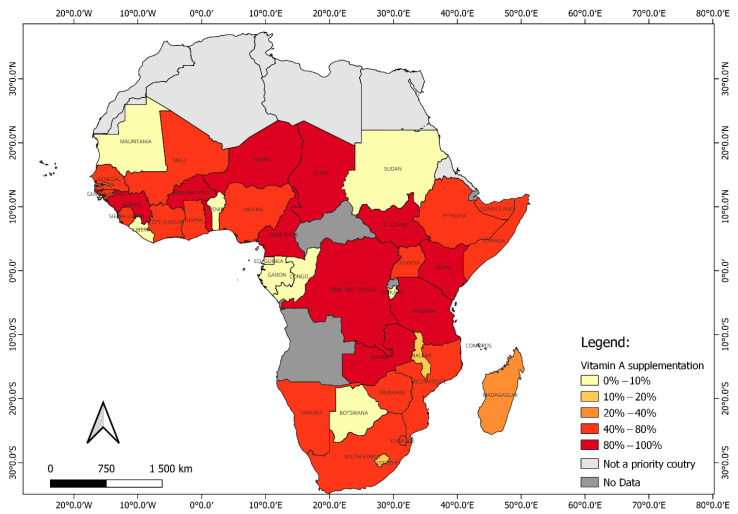
National coverage of VAS programs in sub-Saharan Africa. Despite relatively high coverage in many countries, including Mozambique, vitamin A deficiency remains widespread, illustrating the limitations of supplementation as a sole strategy (adapted from UNICEF data—URL: https://data.unicef.org/topic/nutrition/vitamin-a-deficiency/#data, accessed on 18 April 2024).

Vitamin A supplementation (VAS) remains the cornerstone strategy to address VAD, providing high-dose capsules to children aged 6–59 months. These programs demonstrated significant success in reducing child morbidity and mortality [7,8]. However, the benefits were short-lived, typically lasting only three months [9], and their long-term sustainability was jeopardized by dependence on external donor funding, logistical challenges in maintaining regular distribution, and inequitable coverage between rural and urban populations [10,11,12]. Furthermore, campaign-based supplementation often fails to reach marginalized communities [13,14], leaving persistent gaps in coverage. Recent global nutrition funding trends showed declining support for standalone VAS programs as donors prioritize integrated food system approaches [15], underscoring a growing consensus on the need for sustainable, food-based strategies to complement VAS. As shown in Figure 1, national coverage of VAS programs is relatively high across much of sub-Saharan Africa, including Mozambique, yet these programs have not eliminated VAD. This mismatch reflects the short-term nature of supplementation and the difficulty of reaching all children, further highlighting the importance of food-based strategies.

Within this context, mangoes (*Mangifera indica* L.) are particularly relevant, as they are widely cultivated and consumed in Mozambique and constitute an important seasonal source of provitamin A carotenoids, predominantly β-carotene [16,17,18]. Depending on cultivar and ripeness, β-carotene content ranges from 0.55 to 3.21 mg per 100 g of fresh fruit [19,20]. The main pro-vitamin A carotenoids found in mango are β-carotene, β-cryptoxantin and α-carotene [21]. Their concentration varies depending on the cultivar and the specific part of the fruit (see Table 1). β-Carotene (C_40_H_56_) is a highly unsaturated hydrocarbon and the main precursor of vitamin A (retinol). Its degradation occurs primarily through oxidative, thermal, and photochemical reactions, leading to the formation of apo-carotenals, apo-carotenones, and various volatile compounds that affect both nutritional and sensory quality.

Oxidative degradation is initiated by reactive oxygen species (ROS) or singlet oxygen (^1O_2_), which attack the conjugated double-bond system of β-carotene. The products of this reaction are β-apo-carotenals (C_9_–C_27_), β-apo-carotenones, and cleavage products such as β-ionone, dihydroactinidiolide, and β-cyclocitral. In other words, when an oxidative degradation occurs, there is autoxidation under atmospheric oxygen. This reaction is usually catalyzed by light, heat, metal ions, and peroxides, resulting in color loss and a reduction in vitamin A activity [22,23].

Thermal degradation is activated by high temperatures that accelerate isomerization of β-Carotene from all-trans β-Carotene to cis β-Carotene forms, and oxidative cleavage. The products of thermal degradation are the 9-cis, 13-cis, and 15-cis isomers, followed by smaller volatile breakdown molecules (e.g., β-ionone). When this degradation occurs, there is reduced provitamin A activity and altered bioavailability [24,25].

Photochemical degradation is caused by exposure to light (especially UV and visible wavelengths), which promotes the generation of singlet oxygen, leading to oxidative cleavage. The products of this degradation are the β-apo-carotenals, β-ionone, and other carbonyl-containing volatiles, which are caused by light intensity, wavelength, and presence of photosensitizers (e.g., chlorophylls) [26].

Finally, the enzymatic degradation (Biological Cleavage) is catalyzed by β-carotene 15,15’-dioxygenase (BCO1) and β-carotene 9’,10’-dioxygenase (BCO2) enzymes, yielding retinal also known as vitamin A aldehyde, and apocarotenoids or β-apo-carotenoic acids. This reaction is relevant for physiological conversion pathway in mammals and plants [27].

The bioaccessibility of β-carotene has been estimated at 24–39% [19,28], corresponding to a variable contribution to the recommended dietary allowance (RDA) for vitamin A, depending on the β-carotene content of the mango and serving size [29]. This makes mangoes a promising dietary contributor to reducing VAD, particularly in rural households with limited access to animal-source foods. In Niger, for instance, mangoes accounted for nearly half of children’s vitamin A intake during the harvest season [30].

Yet, mangoes are highly perishable, with postharvest losses estimated at 20–40% in sub-Saharan Africa due to their short ripening season and lack of postharvest infrastructure [31,32]. These losses reduce their year-round availability and limit their contribution to nutrition security [16,33]. Postharvest processing technologies, such as drying, have therefore been proposed to extend the shelf life of mangoes while retaining their nutritional quality.

Traditional open sun drying, though widely practiced, is associated with contamination risks, uncontrolled drying conditions, and nutrient degradation [34,35,36]. In contrast, improved solar drying systems—including enclosed and hybrid dryers—offer higher drying efficiency, better product quality, and greater protection against pests, rain, and dust. These systems also reduce contamination risks and enhance the retention of nutrients such as β-carotene, ultimately leading to longer shelf life and improved food safety [36,37,38]. However, research on the use of improved solar drying for mangoes in Mozambique, and more broadly across Africa, remains scarce [39]. Most existing studies emphasize dryer performance rather than nutritional outcomes, highlighting a critical research gap [31].

This review aims to investigate the potential of improved solar drying technologies to preserve β-carotene in mangoes and support long-term strategies for reducing vitamin A deficiency (VAD) in Mozambique. Specifically, it pursues two complementary objectives:

- Literature review: To synthesize scientific evidence on solar drying of mangoes, focusing on β-carotene retention, drying efficiency, and nutritional outcomes; and
- Qualitative field component: To analyze insights from district health directors in three districts of Inhambane Province, exploring local practices in fruit consumption and assessing opportunities to integrate solar drying into community-level nutrition strategies.

By combining insights from district health directors with evidence from the literature, this evaluation assesses solar drying not only as a technical solution but also as a nutrition-sensitive intervention that complements supplementation programs, reduces postharvest losses, and improves year-round availability of vitamin A-rich foods.

**Table 1:** Distribution of carotenoids in different parts of mango (μg/g).

Cultivar	Carotenoid	Pulp	Peel	Kernel	References
General	β-carotene	6.4 fw	13.1 fw	0.09 fw	[21,40]
	β-cryptoxanthin	0.1 fw	6.0 fw	nd	
	α-carotene	0.09 fw	nd	nd	
Bassignac	β-carotene	41.38 fw	nr	nr	
Arka Anmol	β-carotene	nd	130.1 fw	nr	
Janardhan	β-carotene	nr	7.4 fw	nr	
Badami	β-carotene	32.1 fw	nr	nr	[19]
	β-cryptoxanthin	3.5 fw	nr	nr	
	α-carotene	0.7 fw	nr	nr	
Mallika	β-carotene	28.5 fw	nr	nr	
	β-cryptoxanthin	2.9 fw	nr	nr	
	α-carotene	0.65 fw	nr	nr	
Ataulfo	β-carotene	443 ± 53 dw	604 ± 52 dw	nr	[41]
Keitt	β-carotene	10.8 ± 0.3 dw	8.8 ± 0.8 dw	0.1 ± 0.0 dw	[42]
	α-carotene	nd	1.1 ± 0.0 dw	nd	
Bambagan	β-carotene	200.4 ± 10.1 fw	130.9 ± 2.8 fw	nr	[43]
	β-cryptoxanthin	11.8 ± 0.1 fw	6.0 ± 0.01 fw	nr	
	α-carotene	79.6 ± 15.3 fw	42 ± 1.4 fw	nr	
Tommy Atkins	β-carotene	6.4 fw	nr	nr	[21]
	β-cryptoxanthin	0.1 fw	nr	nr	
	α-carotene	0.09 fw	nr	nr	
Tommy Atkins	β-carotene	6.0 ± 1.0 dw	8.3 ± 0.7 dw	0.1 ± 0.0 dw	[42]
	α-carotene	nd	0.8 ± 0.7 dw	nd	
Tommy Atkins	β-carotene	4.86 ± 0.01 dw	2.78 ± 0.05 dw	0.50 ± 0.01 dw	[44]
	β-cryptoxanthin	2.72 ± 0.04 dw	nd	nd	
Tommy Atkins	β-carotene	18.59 ± 6.12 dw	nr	nr	[45]
	β-cryptoxanthin	1.21 ± 0.61 dw	nr	nr	
	α-carotene	1.71 ± 1.32 dw	nr	nr	

nr = not reported in the cited source, nd = not detected, dw = dry weight, fw = fresh weight.

## 2. Materials and Methods

### 2.1. Geographical Area of the Study

**Figure 2:**
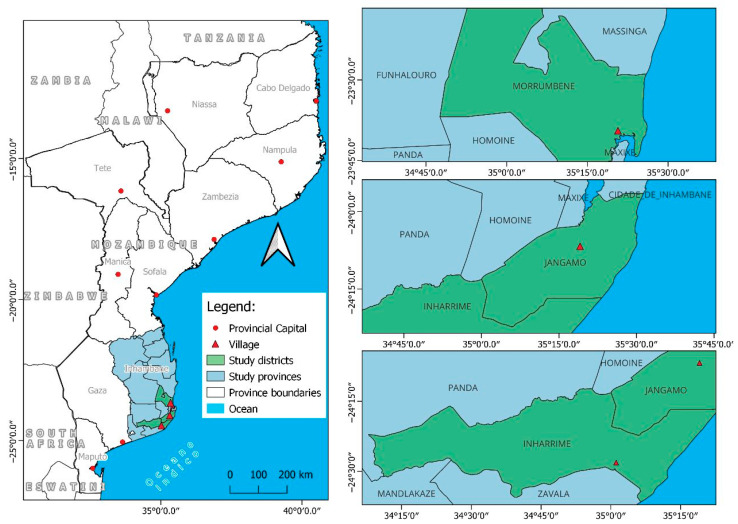
Map showing Mozambique, the Inhambane province, and the districts where the interviews were conducted. Source: the original map was produced in QGIS Desktop 3.28.0, with legend and colour mapping from the author.

Mozambique, located in the southeastern region of Africa, has 10 geographic provinces, and mango is produced in all of them. This study focuses on the Inhambane province (Figure 2, left panel), an area known for high mango production. Within Inhambane, three districts—Morrumbene, Jangamo, and Inharrime—were selected for fieldwork because of their relevance to both mango production and nutrition programming (Figure 2, right panel).

### 2.2. Literature Review

A review of scientific literature was conducted, involving the collection, analysis, and synthesis of existing published studies that had categorized and described various solar drying technologies used in an African context. To achieve this, the authors conducted structured searches using databases such as Google Scholar, Web of Science, and Scopus to identify studies focused on solar drying technologies, particularly as applied to mango preservation in Africa, using the following keywords and combinations: ‘solar drying’, ‘sun drying’, ‘solar dryers’, along with ‘mango fruit’, ‘fruit’, ‘Mozambique’, and the names of specific African countries. The literature search yielded 81 records. From this number, duplicates were removed, and a total of 55 records remained. The titles, abstracts, and keywords of the 55 remaining records were evaluated for relevance, and a total of 21 articles were selected and used in this study. Through this process, data on dryer types, terminology, design adaptations, and performance characteristics were extracted and compared. These results were then synthesized into comparative tables (Tables 2–4) to highlight structural and functional differences across systems, and to assess their reported potential for vitamin A retention.

### 2.3. Interview with Local Health Representatives

Semi-structured interviews were conducted with the directors of district health services from each of the three districts in Inhambane Province. As there is only one health director per district, all three directors were included, ensuring comprehensive coverage of district-level perspectives on health and nutrition coordination. The sampling rationale was therefore purposive and exhaustive, targeting individuals with direct oversight of nutrition-related programs and health interventions.

The interview guide, provided as Supplementary Materials, contained open-ended questions focusing on the prevalence of vitamin A deficiency (VAD), the role of fruit consumption—particularly mangoes—in improving nutritional outcomes, and community practices related to fruit intake. It also explored ongoing initiatives promoting mango consumption as part of a healthy diet, the economic importance of mango sales for food security, and the effectiveness of nutrition education and supplementation programs in addressing malnutrition.

Interviews were conducted in person, allowing for in-depth discussion and clarification. Participants were fully informed about the study’s objectives, procedures, and the voluntary nature of their participation before providing written informed consent. Ethical approval was obtained from the Eduardo Mondlane University’s ethics committee in Mozambique, and additional ethical measures included maintaining confidentiality and anonymizing all responses during analysis.

Responses were analyzed qualitatively to identify recurring themes, opportunities, and gaps in linking mango consumption with nutrition outcomes.

## 3. Results

### 3.1. Insights from the Literature—Solar Drying of Mango in Africa

Postharvest losses of mangoes in sub-Saharan Africa, estimated at 20–40%, undermine their potential as a sustainable source of provitamin A. Solar drying has been increasingly promoted as an alternative to open sun drying, offering faster drying times, improved microbial safety, and better nutrient retention. Given Africa’s high burden of vitamin A deficiency, evaluating solar drying specifically for its role in preserving β-carotene is critical.

In Mozambique, fan-assisted solar dryers (Direct fan (DF), Indirect fan (IF)) reduced drying time by about 40 h compared to open-sun drying (OS), while maintaining microbial safety and offering income opportunities for smallholder farmers [39]. In Tanzania, improved and indirect solar dryers preserved the sensory and nutritional quality of mangoes and pineapples more effectively than traditional methods, due to mild drying temperatures (~40 °C) and improved airflow design [46]. Similarly, a solar greenhouse tunnel dryer achieved safe moisture levels (<12%) within 11–15 h, with insulation and airflow management critical for sustainability [47].

Solar drying systems reported in the literature generally fall into three main categories: direct exposure, indirect, and mixed-mode (or tunnel) designs. Examples include the Open Sun Dryer (OSD) and Direct Flow dryer (DF) for direct exposure; the Improved Solar Dryer (ISD) and Cabinet Direct Dryer (CDD) for indirect systems; and the Cabinet Mixed-Mode Dryer (CMD), Tunnel Dryer (TD), and Hybrid Indirect Passive Dryer (HIP) for mixed-mode configurations. These categories mainly differ in the degree of temperature/airflow control and protection from contamination, which are critical factors for drying speed, microbial safety, and nutrient retention. However, inconsistent terminology across studies (e.g., similar designs labeled DF vs. direct solar dryer (DSD), or “conventional” vs. “cabinet”) complicates comparison and limits standardization. In this study, the OSD represents traditional open-air sun drying with minimal protection, whereas the term solar dryer refers broadly to enclosed systems that utilize solar energy more efficiently. The wide range of designs reported in African mango studies—including direct, indirect, mixed-mode, tunnel, and greenhouse configurations—are summarized in Table 2, which compiles terminology and design descriptions, and Table 3, which outlines their reported applications.

Several studies also report results from electric drying methods (e.g., convective ovens, fluidized bed, freeze, or tray drying) as benchmarks. These are included in Table 3 to contextualize solar drying performance, though they represent controlled conditions that may not reflect the affordability or scalability of solar technologies in African contexts.

**Table 2:** Reported solar dryer designs for mango in Africa. Original author terms and construction details are shown.

Dryer Type	Brief Description of the Drying System	References
DF	Consisted of a chamber made of wood and elevated 1.2 m above the ground with wooden stands. The chamber contained fans to ensure air circulation and was covered with a transparent plastic sheet to ensure the cleanliness of the dried mangoes, which were exposed directly to the sun. The fans were activated by a solar panel connected to the system.	[39]
DnoF	The 1.2 m elevated cabinet was fabricated with two corrugated metal sheets and covered with a transparent plastic sheet. Mangoes were exposed directly to the sunlight. This setup had no fans, and no solar panel connected to the system.	[39]
IF	Same as DF but with an additional wooden board placed over one-half of the collector, shielding the mangoes from direct sunlight exposure.	[39]
Open Sun dryer (OSD)	Consists of a tarpaulin enclosed in a perforated white drying mesh, elevated 1.5 m above the ground using wooden stands. This setup helps prevent dust contamination during the drying process, ensuring the cleanliness and quality of the dried mango.	[46]
Conventional Solar Dryer (CSD)	Made of a dryer enclosed with non-perforated greenhouse plastic polyethylene UV-treated material (Gt4 gauge) that screens ultraviolet (UV) solar thermal radiations.	[46]
White-Cloth Shade (WCS)	Consisted of a white transparent plastic bucket covered with a clean white silk cloth. The mango samples were placed inside the white transparent plastic buckets and then covered with white silk cloth to filter sunlight and simulate shade drying.	[46]
Black-Cloth Shade (BCS)	Same as WCS but, in this case, the white transparent plastic bucket is covered with a black silk cloth to simulate shade drying.	[46]
Improved Solar Drying (ISD)	Consisted of an indirect solar dryer composed of a drying cabinet enclosed with a greenhouse plastic polyethylene material that screens UV radiation and maximizes the retention of absorbed solar thermal energy. These modifications and design enhanced the ISD’s efficiency and effectiveness, improving the drying process while maintaining the quality of the dried produce.	[46]
Cabinet direct Dryer (CDD)	Consisted of a drying unit or a cabinet covered with UV-stabilized visqueen sheets. The mango slices to be dried were placed in trays within the cabinet.	[48,49]
Cabinet mixed mode Dryer (CMD)	Made with two parts: an energy absorber (black painted collector) and a drying unit or a cabinet, covered with UV-stabilized visqueen sheets. The mango slices to be dried were placed in trays within the cabinet.	[48,49]
Tunnel Dryer (TD)	Fabricated with two parts: an energy absorber (black painted collector) and a drying tunnel, covered with UV-stabilized visqueen sheets. The tunnel was equipped with a small fan to provide the airflow for heat circulation and moisture removal from the dryer. The samples were laid in a single layer on a wire mesh within the tunnel.	[48,49,50]
Greenhouse solar dryer	It consisted of a greenhouse walk-in solar dryer covered with a transparent polyethylene exterior, supported by wooden framing and a cemented floor. It combines greenhouse design principles with solar drying techniques to create a controlled environment for efficient drying. The mango slices were arranged on perforated trays in single layers placed on shelves without overlapping.	[51,52]
Indirect solar dryer (ISD)	It is a mixed-type collector combining a corrugated iron absorber and a porous aluminium mesh absorber.	[53]
Direct solar dryer (DSD)	Made of four rectangular trays, supported by a metal framework. Each tray has a wooden frame with a nylon net bottom to facilitate air flow. The trays are stacked with the top tray directly exposed to solar irradiance. The dryer’s sides are covered with a nylon net to prevent contact between the drying product and the external environment, allowing for efficient air circulation.	[31]
Hybrid Indirect Passive (HIP)	Consists of a rectangular solar collector plate with metallic concentrators made of 15 steel tubes that absorb and retain solar heat. The collector is covered with transparent PVC plastic to maximize solar radiation absorption, and the bottom layer is lined with silver plastic to reflect heat towards the tubes. The drying cabinet is covered with UV-treated greenhouse plastic to enhance internal warming through the greenhouse effect. The operation involves pre-heating incoming air in the metallic concentrators, which rises naturally to the drying cabinet. The greenhouse effect further warms the air, and excess heat, vapor, and moisture are managed through a chimney to prevent over-drying.	[54]
Solar Photovoltaic and Electric (SPE)	Consists of a system with two 60 W solar panels connected to four batteries to ensure continuous drying. A 12 V suction fan regulates air circulation, operating at 50% capacity with a preset air velocity of 15 m/s. An auxiliary electric backup system provides drying power during cloudy conditions or at night. The automatic control system, including a thermocouple and charge controller, manages drying conditions and ensures consistent drying despite temperature fluctuations.	[54]
Natural convection solar dryer (NCSD)	Consists of a structure, like a solar chimney, that enhances air circulation for improved drying rates. The drying occurs in an enclosed or semi-enclosed environment, using natural airflow (convection) enhanced by design elements like the solar chimney. This setup speeds up the drying process, protects products from contaminants, and ensures more uniform and consistent drying.	[55]
Natural convection solar tunnel dryer (NCSTD)	The natural convection solar tunnel dryer is a modular system that uses solar energy to create buoyancy-driven airflow for drying. Air is heated in a flat-plate collector, passes through a drying unit with wire mesh trays to absorb moisture, and is reheated in a chimney before being expelled. The dryer, constructed from galvanized iron and insulated with Styrofoam, is elevated and designed to optimize drying through both convective heat transfer and direct radiation, ensuring high drying rates.	[56]

A review of published scientific work on the use of solar drying to dry mangoes, using the above-described drying systems, is reported in Table 3. The main conclusions with regard to microbial analysis and sensory evaluation are also included in the table, according to each study. This review is limited to Africa, as this continent has the highest prevalence of vitamin A deficiency among children, followed closely by Southeast Asia. The deficiency is primarily caused by limited access to vitamin A-rich foods and poor dietary diversity, worsened by poverty and food insecurity.

Despite the increasing interest in solar drying as a method for food preservation, significant gaps remain in the research concerning its impact on vitamin A preservation. The literature search, which was not confined to a specific time frame, yielded results only from 1989 onward. Between 1989 and 2025, a total of 21 scientific papers were published on the solar drying of mangoes in Africa. As Table 3 shows, only about 40% of these assessed vitamin A outcomes, with the majority focusing instead on dryer performance (e.g., thermal efficiency, drying rates, and final moisture content). Moreover, research is unevenly distributed: Mozambique, despite high VAD prevalence, has only one published study [39], while most vitamin A-focused evidence comes from Kenya and Senegal. The existing papers, while valuable, mainly focused on analyzing parameters related to different types of solar dryers, including their thermal performance, physical characteristics, and water content of mango after drying.

**Table 3:** Review of the research conducted on solar drying of mango in Africa.

Country	Solar Drying Technology	Main Conclusion	Focus on Pro-Vit. A	Reference
Burkina Faso	Indirect solar dryer.	A model of thin-layer drying of mangoes was created. Three “typical days” of drying were necessary to reach the water content of conservation (13.79%). The maximum drying efficiency was 35.04%, with a maximum drying rate of 0.18 g kg^−1^ s^−1^.	No	[53]
Burkina Faso	DSD.	A model of thin-layer drying of mangoes was created. Four days of drying were necessary to reach a final water content of 24.83% db for the Amelie cultivar and 66.32% db for Brooks. Drying efficiency ranged from 0 to 34%. Drying rates were 0.150 g kg^−1^ s^−1^ and 0.153 g kg^−1^ s^−1^.	No	[32]
Cameroon	Direct Open Sun and Electric Oven.	The solar drying gave the same retention rate (56%) of nutrients (vitamin C, reducing sugars, and water-soluble extracts) but took longer to dry than electric drying.	No	[57]
Democratic Republic of the Congo	Oven dryer and Solar dryer.	Solar drying produced the same results for mineral contents (11 mg/L of Ca, 10.64 mg/L of Mg, and 0.08 mg/L of Fe) and vitamins A, D, and E, as oven drying. Reported similar or higher nutrient concentrations in dried samples, though this increase likely reflects concentration effects from water loss rather than true nutrient enhancement. For solar-dried mangoes, fungal contamination becomes a concern after 90 days of storage, requiring proper storage solutions. Sensory evaluation found that oven- and sun-dried mangoes were rated as acceptable; no difference was observed in the attributes of odor, taste, and texture between the solar dryer types. Regarding the color attribute, the sun-dried mangoes appeared browner due to non-enzymatic heat-induced browning.	Yes	[58]
Democratic Republic of the Congo	Sun-dryer and oven- dryer.	The sun drying of mangoes benefits human health due to nutrient retention and the potential for increased vitamins and minerals. Sun-dried mangoes retained more vitamin C (205 mg/100 mL after sun drying compared to 64.8 mg/100 mL after oven drying) and calcium (11 mg/L over 9 mg/L). Oven-dried mangoes retained more vitamin B6 (139 mg/100 mL over 69.7 mg/100 mL). Magnesium and Iron did not differ significantly between the two drying technologies. Pro-vitamin A was identified in both oven-dried and solar-dried mangoes, but comparisons are not reported. Reported nutrient ‘retention’ should be interpreted cautiously, as apparent increases often reflect concentration effects from water loss rather than true preservation. The microbial analysis (mesophilic, osmophilic and fungal germs; salmonella, staphylococcus and coliforms) showed no contamination on the samples. Sensorial analysis showed no differences between dryers in terms of odor, taste and texture. Samples dried in the sun were browner due to non-enzymatic browning reaction. The sun drying technology is transferable to farmers to reduce post-harvest losses and improve their economic situation.	Yes	[59]
Ethiopia	Solar dryer, tray dryer, freeze dryer, and fluidized bed dryer.	Solar drying scored the lowest vitamin C content (33.18 mg/100 g), with fluidized and freeze drying registering the highest values (41.24 and 41.06, respectively); and the highest phenolic contents (251.12 mg/100 g) when compared to other drying methods. Freeze-drying and fluidized-bed drying methods registered the best moisture content (7.88% and 5.63%, respectively), and these technologies hold the potential to significantly reduce mango postharvest losses after harvest in Ethiopia.	No	[60]
Ghana	Solar drying and oven drying.	Solar drying was superior in preserving fat (0.61%), magnesium (134 mg/kg) and calcium (39.6 mg/kg). Oven drying preserved protein (3.17%), ash (2.60%), carbohydrates (84.25%), iron (6.70 mg/kg) and potassium (396 mg/kg) better. Oven drying is more suitable for drying Keitt mango pulp and combating malnutrition.	No	[61]
Ivory Coast	Natural solar drying.	The higher the mango’s initial moisture content (Wi), the bigger the difference between the temperature of the surrounding air (Ta) and the mango itself (Tp) during drying (DTmax). Mangoes’ drying rate is strongly influenced by their Wi and DTmax. When the drying rate slows down (falling rate period), mangoes exhibit the highest resistance to moisture diffusion compared to other fruits.	No	[62]
Kenya	Sun dryer, solar dryer, and oven dryer.	The oven dryer took 2 h to dry mangoes to a moisture content of 10–12%; the solar dryer took 1 to 2 days to reach the same final moisture content. Blanching before drying resulted in significantly higher beta-carotene retention in dried mangoes compared to sugar dip, ascorbic acid dip, and control treatments across all drying methods. In the “Tommy Atkins” cultivar, it preserved 91%, 76%, and 54% of the original carotenoid content when subjected to oven drying, solar drying, and sun drying, respectively. For the “Ngowe” cultivar, blanching retained 64%, 54%, and 44% of the carotenoid content under the same drying conditions. The carotenoids content after drying could cover for more than 50% of the daily requirement of pro-vitamin A in children and adult women, indicating that, under favorable conditions, dried mango could contribute substantially to daily vitamin A requirements.	Yes	[63]
Kenya	Oven dryer and solar dryer.	The samples in the oven dryer dried 7 h faster than those in the solar dryer. Pre-treating mangoes with a citric acid dip prior to drying helps retain more antioxidants in dried mango products with the oven drying method. Large differences in carotenoid content reported, depending on both drying method and pre-treatment, making it difficult to isolate the effect of dryer type alone.	Yes	[38]
Mozambique	DF, Direct solar dryer with no fan (DnoF), IF, and OS drying.	Solar dryers with fan-assisted setups (DF and IF) dried mango slices about 40 h faster than OS and achieved safe water activity levels of less than 0.6, unlike the non-fan setup (DnoF). Microbial safety was maintained across all methods, with total aerobic plate counts staying within safe limits. Economically, using fan-assisted dryers could generate significant income for smallholder farmers in Mozambique.	No	[39]
Nigeria	OSD, oven drying and solar drying.	Pretreated dried mangoes with honey appeared to protect carotenoids and vitamin C during drying. It took 8 h to reach a final water content of 6%. The average drying temperature in the solar dryer was 41 °C, and the drying rate was 40 g/h. There was no significant difference between the drying methods.	Yes	[37]
Senegal	Greenhouse solar dryer.	Greenhouse solar dryers reduced mango postharvest losses. Initial 3.75 tons of fresh mango resulted in 360 kg of final dried mango, demonstrating that greenhouse drying can preserve β-carotene at levels sufficient to make meaningful contributions to dietary vitamin A intake at scale. It is a promising strategy to combat VAD in French-speaking West Africa.	Yes	[51]
Senegal	Greenhouse solar dryer.	Solar-dried mangoes are highly nutritious, retaining essential nutrients like beta-carotene (3.8 mg/100 g), vitamin C (22 mg/100 g), and iron (1.1 mg/100 g), crucial for addressing dietary deficiencies, particularly in children. Solar drying is an affordable, accessible, and effective preservation method that aligns with local preferences. It offers significant potential to reduce food waste, improve food security, and create economic opportunities. This technology is well-suited to tropical environments where sunlight is plentiful year-round. 8 trained panelists rated all solar-dried mangoes “Ok to good”, with mean scores ranging from 2.7 to 3.7 on a 1–5 scale for brightness, sweetness, and overall acceptability. Pretreatments, cultivar, and storage did not affect any of the sensorial attributes. Dried mango-based desserts rated “good–excellent” by Senegalese consumers, in a categoric hedonic scale with four descriptive levels: “bad,” “good,” “very good,” and “excellent”.	Yes	[52]
Sudan	NCSD.	A functional prototype of a solar dryer with a maximum collector area of 1.03 m^2^ was built. Mango slices were successfully dried in 2 days drying period (20 h) to a moisture content of 10%.	No	[55]
Tanzania	OSD, CSD, WCS, BCS and ISD.	Sensory quality attributes (colour, texture, flavor, and aroma) were better retained using the ISD dryer than traditional solar dryers. The same was observed with the nutritional content, reporting very high carotenoid retention in improved systems, though such near-complete preservation (>95%) should be interpreted cautiously. Sensory evaluation involving 15 trained panelists indicated that solar-dried mangoes were generally acceptable in terms of taste, aroma, color, and overall appearance, with overall scores ranging between 5.0 and 8.0 on the nine-point hedonic scale. The CSD and ISD dryers achieved higher sensory scores across these attributes (≈ 7.5 to 8.0) compared to OSD, WCS, and BCS (≈ 5.0 to 6.0). Dried mangoes were rated lower in overall acceptability due to limited consumer exposure and familiarity with solar-dried mango products.	Yes	[46]
Tanzania	CDD, CMD, and TD solar drying methods.	The drying process significantly impacts dried mangoes’ total phenolic content and antioxidant activity. Compared to CDD and CMD, TD leads to less total phenolic content loss (6–16% vs. 17–42%), indicating better preservation of antioxidant properties in dried mangoes.	No	[48]
Tanzania	CMD and TD.	Solar drying benefits dried fruits by improving their quality, reducing harmful microbes, and extending their shelf life. TD samples had lower moisture content (11.2%), water activity (0.49), and fungal loads (0.8 log CFU/g) compared to cabinet-dried fruits (with moisture content 12.5%, water activity 0.55, and fungal loads 1.2 log CFU/g). Solar drying reduced microbial loads, with total plate counts decreasing from 4.1–4.2 to 3.4–3.9 log_10_ CFU/g, particularly in tunnel-dried samples. Products remained microbiologically safe due to low moisture and good hygienic practices, with no coliforms detected, although yeast and mold slightly exceeded 3 log_10_ CFU/g.	No	[49]
Tanzania	CDD and TD.	Over 65% of the nutrients (fat, ash, fibre, and protein), 87–99% of sugars, and 71–80.1% of organic acids were retained, with TD being more effective than CDD (except for sugars). Solar drying is a viable method for mango preservation with minimal negative effects on its nutritional value.	No	[50]
Uganda	HIP, SPE & OSD.	SPE dryer achieved the fastest drying rate (0.209 g/min) and highest efficiency (84.3%) among the tested methods. OSD method had the slowest drying rate (0.074 g/min) and lowest efficiency (35.1%). SPE drying method took 10 h to dry the samples, while HIP took 18 h, and the slowest drying method (OSD) took 30 h to dry the samples.	No	[54]
Zambia	NCSTD.	The drying process successfully reduced the moisture content of mango slices to the desired level of 13% in 9.5 h. Performance was sufficient to drive the flow of air through the dryer.	No	[56]

As shown in Table 4, reported drying durations for mango varied widely, from as little as 2 h in oven drying at 70 °C to up to 4 days under direct open-sun exposure. The initial moisture content was typically 80–85% (wet basis), decreasing to 10–15% after drying. Solar dryers operating between 45 °C and 55 °C provided faster and more uniform drying than open-sun methods (23–29 °C), while oven drying achieved the lowest final moisture content (8–10%). Controlled drying systems (such as oven, solar, and fluidized-bed dryers) consistently reduced drying time and achieved lower final moisture content compared with traditional open-sun drying. However, few studies reported key process parameters such as airflow rate, relative humidity, irradiance, or drying efficiency, highlighting the need for standardized reporting of drying conditions to enable meaningful comparison of performance across dryer types. 

Table S1. Quantitative summary of β-carotene or vitamin A retention (µg/100 g), reported in the Supplementary Materials, summarizes the reported mean ± SD retention rates of β-carotene in dried mango, organized by dryer type and pretreatment method. It also indicates the RDA for the highest and lowest β-carotene retention, under two categories of dryers (oven dryers and solar dryers), assuming a reasonable intake of 20 g of dried mango for children aged 3 to 6 years old. The values were taken from studies that explicitly reported β-carotene or vitamin A retention outcomes.

### 3.2. Impact of Solar Drying on Pro-Vitamin A Content Retention

Solar drying plays a critical role in carotene retention, but outcomes vary depending on drying methods, pre-treatments, cultivar, and environmental conditions. Thin-layer drying models and fan-assisted systems, as reported in Burkina Faso [31,53] and Mozambique [39], promoted uniform moisture reduction, which in turn supports nutrient preservation.

Traditional open sun drying, also called by different authors as OS or just as sun drying, often resulted in higher nutrient losses, while advanced systems (indirect, greenhouse, photovoltaic-assisted) generally achieved better retention [50,51,52,55]. Environmental conditions also influenced outcomes: high drying temperatures can degrade heat-sensitive nutrients, while low temperatures or high humidity can increase microbial risk [49,51,52,55]. For a comprehensive overview of research conducted on solar drying of mango in Africa, refer to Table 3.

In Kenya, studies on Tommy Atkins and Ngowe mango cultivars illustrated differences between oven, solar, and sun drying. Oven drying preserved ~91% of carotenoids in Tommy Atkins, compared to 76% with improved solar dryers and 54% with sun drying. Ngowe showed lower values at 64%, 54%, and 44%, respectively [58,63].

Pre-treatments can enhance carotene retention. Honey and ascorbic acid dips in Nigeria protected carotenoids during drying, with honey-treated mangoes yielding up to 5922 µg β-carotene/100 g–enough to meet ~100% of children’s daily vitamin A needs [37]. Steam blanching in Kenya also improved retention, though at lower levels (4100–5500 µg/100 g) [38].

Using the standard conversion factor (12 µg β-carotene = 1 µg RAE [29,64,65]), 100 g of dried mango can provide ~60–100% of daily vitamin A needs for children and ~50–70% for women, depending on cultivar and pre-treatment. Greenhouse solar dryers in Senegal, for example, retained 3.8 mg/100 g of β-carotene, equivalent to ~20–31% of adult RDA [29,52]. Reports of near-complete retention (>95%) from Tanzania [46] should be interpreted cautiously, as they likely reflect controlled experimental settings rather than farmer conditions. Within the studies conducted on the solar drying of mango in Africa, it was noted that oven dryers with pretreatments yielded higher β-carotene levels (Table S1 in the Supplementary Materials). Still, they require stable electricity and precise control, which limits their use in rural areas. Solar dryers are more practical and affordable, although they are highly dependent on weather conditions, making replication difficult. Environmental variability can be used to explain the differences in β-carotene levels among samples dried under solar conditions. The differences in mango cultivars also influence nutrient retention after drying; therefore, selecting cultivars suited for both taste and nutrient preservation is essential. Limited access to specific cultivars may hinder the replication of high-value results. Solar drying with lemon juice pretreatment is the most feasible method for rural areas. Lemon juice is inexpensive, readily available, and helps retain carotenoids naturally. Improved dryers, hygiene, and timing can enhance solar drying efficiency.

Beyond these nutritional insights, closer examination of the available studies also reveals methodological and contextual limitations that constrain their applicability.

While the overview above highlights study-specific findings, Table 4 consolidates this evidence by mapping different dryer types against reported β-carotene retention levels and their potential relevance for VAD reduction. This comparison helps visualize which technologies hold the greatest promise from a nutritional perspective.

While Table 3 and Table 5 summarize the technical and nutritional potential of solar drying, the interviews illustrate why these technologies have not yet translated into nutritional outcomes in Inhambane: mangoes are primarily sold, preservation knowledge is minimal, and VAD is seen as controlled through supplementation. Bridging this gap requires strategies that align technical solutions with community-level practices and perceptions.

### 3.3. Existing Gaps in Research on Solar Drying and Its Impact on Vitamin A Preservation

#### 3.3.1. Geographical Gaps

**Figure 3:**
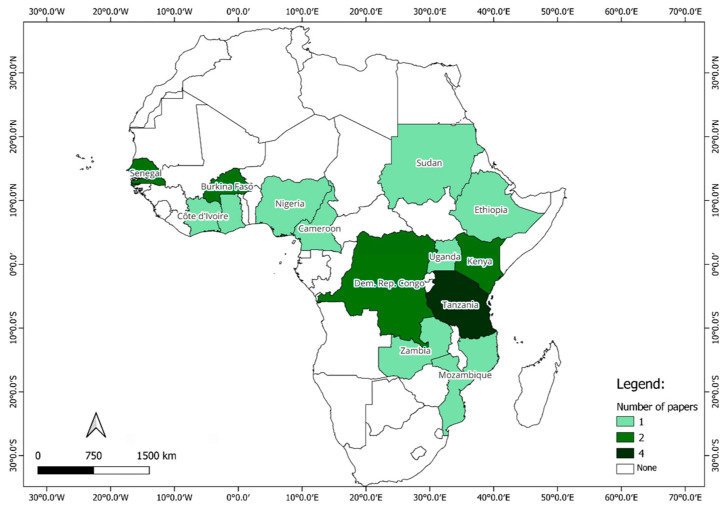
Distribution of published studies on solar drying of mango in Africa (1989–2025). Data are drawn from the author’s review of 21 peer-reviewed studies published between 1989 and 2025. Approximately 40% of these studies examined vitamin A or β-carotene retention, while the remaining studies focused on dryer performance parameters such as thermal efficiency, drying rate, and final moisture content. Research outputs are concentrated in a few countries (e.g., Kenya, Tanzania, Senegal), whereas high-need countries such as Mozambique have very limited evidence, highlighting a mismatch between nutritional needs and research attention (author’s review).

While Table 3 highlights the scarcity and uneven distribution of vitamin A-focused studies, further limitations emerge when examining study design and methodology. As illustrated in Figure 3, research outputs are concentrated in a handful of countries (e.g., Kenya, Tanzania, Senegal), whereas high-need countries such as Mozambique have produced only one published study [39]. This mismatch underscores how current research efforts are not aligned with nutritional needs.

Despite Mozambique’s significant mango production and high VAD burden (69% prevalence among children), evidence on solar drying remains minimal. Tanzania has the highest number of reports, but still only four studies, while most other countries with high VAD prevalence have none. This scarcity illustrates a critical gap: despite the abundance of mangoes, little evidence exists on how solar drying can enhance vitamin A consumption, indicating an underexplored opportunity for a sustainable, food-based intervention to improve year-round vitamin A intake.

#### 3.3.2. Methodological Gaps

Most available trials are small-scale, short-term, and limited to single cultivars, making it difficult to generalize findings across Africa’s diverse mango varieties and agroecological zones. Inconsistent carotenoid assay methods and reporting units further hinder cross-study comparison and dietary impact estimation. Early work, such as Rankins et al. (1989) [52], showed that solar-dried mangoes could retain significant provitamin A activity but was restricted to three varieties and a single dryer design. More recent studies (e.g., [36]) demonstrated that pretreatments like honey or ascorbic acid could enhance β-carotene retention, yet these experiments were often conducted under laboratory conditions with small sample sizes and limited cultivars, raising questions about real-world applicability. To strengthen the evidence base, future research should use standardized analytical protocols, include multiple sites and cultivars, and evaluate dryers under practical farmer-managed conditions.

#### 3.3.3. Contextual Gaps

Beyond technical performance, very few studies integrate nutrient retention data with adoption dynamics, costs, or household practices. Evidence is scarce on whether households would consume dried mango or prioritize it for sale, on how cultural preferences shape acceptability, and on whether communities view dried mango as a viable complement to supplementation programs. Economic analyses of dryer affordability, maintenance, and scalability are also limited. Without this contextual understanding, it is difficult to assess whether solar drying can realistically improve vitamin A intake at household or population level.

Addressing these geographical, methodological, and contextual gaps will require coordinated, multidisciplinary field trials across multiple mango-producing countries, coupled with harmonized laboratory protocols and research on consumer adoption. Such efforts would provide reliable estimates of the contribution of solar-dried mango to children’s vitamin A intake and inform scaling strategies. By preserving carotenoids and extending mango availability beyond the harvest season, solar drying has the potential to serve as a sustainable, food-based intervention to enhance year-round vitamin A intake.

### 3.4. Interview Findings

Interviews with directors of health services in Morrumbene, Jangamo, and Inharrime confirmed the importance of mango in local diets but revealed that most harvests are sold, with minimal preservation beyond jams production. Providers perceived that fewer than 10% of children currently suffer from micronutrient deficiencies, a much lower figure than national survey data suggest (69% prevalence of VAD among children 6–59 months) [11]. This discrepancy likely reflects the impact of supplementation programs in their districts but also illustrates the gap between local perceptions and national-level evidence. Providers further cautioned that discontinuation of supplementation campaigns would likely lead to a rapid resurgence in VAD prevalence.

Communities recognize mango’s nutritional value, seasonal scarcity, limited preservation knowledge, and commercial priorities reduce its dietary impact. These findings are summarized in Box 1 and, when read alongside Table 5, illustrate how technical solutions for β-carotene retention must be matched with community-level strategies for adoption.

Box 1 **Community perspectives on mango and nutrition (In the Inhambane province).**

- Perceptions: Mango is recognized as nutritious but primarily valued as a cash crop.
- Practices: Most harvests sold; minimal preservation beyond jams; fresh consumption seasonal.
- Nutrition relevance: Low household consumption despite high local production; children rely mainly on supplementation.
- Policy risk: Health workers warn that stopping VAS could rapidly increase deficiency rates.
- Opportunity: Training in drying/preservation could extend seasonal benefits, complement supplementation, and balance income with household nutrition.
- Implication for dryer choice: Technologies that are low-cost, simple to use, and visibly preserve product quality (e.g., greenhouse or fan-assisted dryers) may have greater chances of adoption.

## 4. Discussion

This study highlights a persistent disconnect between the technical potential of solar drying technologies and their limited nutritional impact in Mozambique. While the literature summarized in Table 2, Table 3, Table 4 and Table 5 demonstrates that improved dryers can preserve carotenoids far more effectively than traditional open-sun methods, adoption in real-world settings remains the decisive barrier (for an extensive analysis of barriers for the adoption of solar dryers, see Viola Salvador et al., 2025 [66]).

A critical finding is that the literature and fieldwork point in different directions. Technical studies emphasized the efficiency of improved dryers: greenhouse and indirect systems could retain >60% of β-carotene, and 100 g of dried mango could, in theory, cover most of a child’s daily vitamin A needs. Fan-assisted dryers further reduced drying times and improved microbial safety, while several studies highlighted the economic opportunities of dried mango as a value-added product. In contrast, interviews in Inhambane revealed that these nutritional and economic benefits are not currently realized at the community level. Mangoes are primarily sold as cash crops, preservation knowledge is minimal, and dried products play little to no role in household diets. The directors of health services continue to see supplementation as sufficient, with dried fruits such as mango largely absent from nutrition programming.

These differences highlight why technical promise alone is insufficient. Nutrient retention and microbial safety do not automatically translate into improved vitamin A intake unless households adopt drying practices. Moreover, the perceived profitability of fresh mango sales discourages investment in drying equipment, while supplementation campaigns reduce the sense of urgency around food-based alternatives. In short, the challenge is not a lack of effective technology, but a gap between the dryer’s performance and community adoption. Bridging this divide requires strategies that make dried mango both economically attractive and culturally acceptable, while embedding it within broader nutrition and agricultural policies.

The technical evidence suggests a hierarchy of relevance. Direct dryers, though inexpensive and simple, showed the lowest β-carotene retention and therefore limited value for reducing VAD. Indirect and greenhouse dryers performed better, with Senegalese studies showing meaningful contributions to dietary intake. Hybrid and photovoltaic-assisted designs hold additional promise by reducing drying time and microbial risks, though nutritional data remained sparse. This indicates that the choice of dryer technology has implications not only for nutrient preservation but also for adoption potential in different contexts.

From a socioeconomic perspective, solar drying could align nutrition with livelihoods by enabling households to allocate part of the harvest for drying and part for sale, creating both income and dietary contributions. Yet without hands-on training, access to affordable equipment, and supportive extension services, households are unlikely to adopt drying at scale, regardless of technical performance.

Programmatically, solar drying should be framed as a complement, not a substitute, to VAS [8]. While vitamin A supplementation (VAS) provides protection for approximately three months [9], a complementary, year-round strategy could combine supplementation with the regular consumption of solar-dried mangoes. Such an approach would align the short-term benefits of supplementation with food-based solutions capable of sustaining vitamin A intake between campaigns. For example, supplementation could be maintained during high-risk periods (e.g., the rainy season, when infection rates and morbidity tend to rise), while solar drying initiatives promote the preservation and household consumption of dried mangoes during the dry and post-harvest months. VAS remains essential for immediate child health protection in high-burden settings [67]. Food-based solutions like dried mango can improve the stability of vitamin A intake over the year and fit well with community diets. Experience with other food-based interventions in Mozambique—such as orange-fleshed sweet potatoes—shows that culturally relevant, locally available options can deliver lasting nutrition gains when paired with supplementation and behavior change support [68].

Priorities follow directly from these insights. First, invest in capacity building (hands-on training in drying and safe storage) and affordable access to improved dryers through extension and local enterprise. Second, embed food-based approaches into national nutrition and agriculture policies so that drying is not a standalone project but part of routine systems. Third, strengthen the evidence base with large-scale, real-world evaluations that measure adoption, household consumption, and nutritional outcomes [69,70]. Finally, explore synergies with complementary interventions, such as fortification and biofortification, to ensure dried mango contributes to a diversified strategy rather than carrying the full burden alone [71].

In summary, solar drying can help transform seasonal abundance into year-round nutritional value, but its contribution to reducing VAD will depend on household adoption and policy integration, alongside the ongoing protection afforded by supplementation.

### Limitations

This study included only three semi-structured interviews, corresponding to the three districts examined in Inhambane province. While the small number of respondents limits the ability to generalize the findings across Mozambique, the interviewees were directors of health services for each district, with direct knowledge of nutrition programming and community practices. As such, their perspectives offer valuable institutional insights into the implementation context. The interviews were not designed to capture household- or individual-level consumption dynamics but rather to complement the literature review by highlighting programmatic realities and perceived barriers from a health system perspective. Because no household- or individual-level data were collected, it was not possible to quantify actual consumption patterns, adoption rates, or vitamin A intake. Moreover, current evidence does not establish any causal link between the adoption of solar drying technologies and improvements in vitamin A status. The findings should therefore be interpreted as indicative of potential pathways rather than demonstrated nutritional effects.

## 5. Conclusions

This review examined the potential of solar drying of mangoes as a complementary strategy to address vitamin A deficiency (VAD) in Mozambique. The findings indicate that while improved dryers can preserve β-carotene and reduce postharvest losses, their nutritional benefits will only materialize if households and communities adopt them as part of everyday practice. At present, mangoes are primarily sold rather than preserved, and dried mango plays little role in local diets. The directors of health services perceive supplementation as sufficient, despite national data showing a persistent high prevalence of VAD among children. This disconnect underscores the need for food-based solutions that are not only technically effective but also socially and economically viable.

The broader lesson is that sustainable nutrition strategies cannot rely on supplementation alone. Supplementation provides vital short-term protection, but it does not address structural vulnerabilities such as dependence on donor funding, logistical challenges in reaching remote households, or the seasonal scarcity of vitamin A-rich foods. Solar drying offers a way to bridge this gap by extending the availability of mangoes throughout the year and providing opportunities for income generation through value-added products.

Moving forward, investments in capacity building, affordable dryer designs, and integration into agricultural and nutrition policies will be critical. Equally important is strengthening the evidence base with large-scale field evaluations that measure not only β-carotene retention but also adoption dynamics and actual dietary contributions. Mozambique’s experience demonstrates both the opportunity and the challenge: abundant mango production coexists with high VAD prevalence, showing that availability does not guarantee nutrition.

By embedding solar drying within broader food system strategies, Mozambique can transform a seasonal fruit into a year-round nutritional resource. In doing so, it can advance both public health and economic resilience, while offering a model for other countries facing similar challenges.

## Figures and Tables

**Table 4:** Dryer types and key drying conditions of studies across Africa.

Dryer Type	Drying Conditions									Reference
	Duration (Days or Hours)	Air Flow (m/s)	Solar Irradiance (MJ/m^2^.Day)	Relative Humidity (%)	Temperature (°C)	Initial Water content (%)	Final Water Content (%)	Drying Efficiency (%)	Drying Rate (g/kg.s)	
Direct Solar dryer (DSD)	4 days	~2.8	15–19	14–40	25–35	Amélie: ~85.7% (wb.) Brooks: ~85.3% (wb.)	Amélie: ~24.8% (db.) Brooks: ~66.3% (db.)	0–34	0.15	[32]
OSD, OD & Solar dryer	8, 6 and 8 h respectively	nr	nr	nr	32, 65 and 41 respectively	nr	nr	nr	nr	[37]
OD (50 and 65) and Solar dryer	(11 h, 7 h) and 18 h respectively	nr	nr	nr	50, 65 °, & nr respectively	nr	nr	nr	nr	[38]
DF, DnoF, IF, and OS dryer	25 h, 74 h, 23 h, and 63 h	nr	19–30	5–70	28–80	84.3 ± 2.0% w.b	nr	nr	1.8, 1.5, 1.9, and 1.02 respectively	[39]
OSD, CSD, WCS, BCS & ISD	10 h	nr	nr	nr	nr	nr	8% db	nr	nr	[46]
CDD, CMD, and TD	3 days	nr	nr	nr	30–55, 25–49, and 60–73 respectively	nr	nr	nr	nr	[48]
CMD & TD	3 days and 2 days respectively	nr	nr	nr	30–55 and 30–73 respectively	nr	nr	nr	nr	[49]
CDD and TD	3 days and 2 days respectively	nr	nr	nr	30–55 and 30–73 respectively	77.8–97 wb	13.9–14.1 db	nr	nr	[50]
Greenhouse solar dryer	24–28 h	nr	nr	nr	nr	nr	nr	nr	nr	[51]
Greenhouse solar dryer	48 h	nr	nr	nr	34–48	nr	9–11 db	nr	nr	[52]
Indirect solar dryer	3 days	nr	~73.44	15–50	35.3–62.6	85.73 wb	16 db	35	Day 1: 0.18, Day 2: 0.13, Day 3: 0.04	[53]
HIP, SPE & OSD	18 h, 10 h, and 30 h respectively	nr, 15, and nr respectively	172.6, 215.8 and 129.4 respectively	nr	27.7, 40.3 and nr respectively	nr	10 db	84.3, 53.7 and 33.8 respectively	~0.209, 0.086 and 0.074 respectively	[54]
NCSD	10 h	2	20	15–51	30	81.4 wb	10 wb	30	0.0007	[55]
NCSTD	12 h	0.04	31.8	6.4–30.8	32.8	85.5 wb	13 wb	12.8	nr	[56]
Direct Open Sun and Electric Oven	27 h	nr	nr	40–68	30–43	74–81 wb	15–23 wb	nr	nr	[57]
Oven dryer and Solar dryer	24 h and +24 h respectively	nr	nr	nr	50 and 22.4–40.7 respectively	88.26 wb	8.25 wb. and 14.17 wb. respectively	26 and 33 respectively	nr	[58]
Oven dryer and Solar dryer	24 h and +24 h respectively	nr	nr	nr	50 and 22.4–40.7 respectively	nr	8.25 wb. and 14.17 wb. respectively	nr	nr	[59]
Solar dryer, tray dryer, freeze dryer, and fluidized bed dryer	nr	nr, nr, nr, and 1.5 respectively	nr	nr	nr, 70, −52, and 50 respectively	80.03 wb	5.63–9.91 wb	nr	nr	[60]
Solar drying and oven drying	24 h and 11 h respectively	nr	nr	nr	36–40 and 60 respectively	80.44 wb	10.42 and 8.26 respectively	nr	nr	[61]
Natural solar drying	nr	nr	nr	nr	nr	84 wb	15–20 wb	nr	nr	[62]
Sun dryer, solar dryer, and oven dryer	3 days, 2 days and 2 h	nr	nr	nr	23–29, 45–55 and 70 respectively	nr	10–12 wb	nr	nr	[63]

nr = not reported, db = dry basis, wd = wet basis.

**Table 5:** Solar dryer types, carotenoid retention evidence, and potential relevance for vitamin A deficiency (VAD) reduction (clarified).

Dryer Type	Key Technical Features	Reported β-carotene Retention (Examples from Literature)	Potential Relevance for VAD Reduction *
Direct solar dryers (DF, DnoF, OSD, DSD)	Food directly exposed to sunlight; minimal control over temperature/airflow.	Kenya: 44–54% retention (Ngowe), 54% (Tommy Atkins) [63].	Limited–nutrient losses can be substantial, but still provide some contribution to dietary vitamin A during harvest season.
Indirect solar dryers (IF, ISD, cabinet dryers)	Heat via collector; mango protected from direct sun; airflow partially controlled.	Kenya: ~54–64% retention with blanching [63]. Tanzania ISD: very high retention reported (up to ~99% [46]), though such near-complete values likely reflect controlled experimental settings and measurement choices, rather than outcomes achievable under typical field conditions.	Moderate to high–improved retention compared to direct dryers; can extend seasonal vitamin A availability if scaled.
Mixed-mode / hybrid dryers (CMD, TD, HIP, NCSTD)	Combine direct + indirect heating; some include fans/solar chimneys.	Tanzania: CMD/TD preserved sugars and organic acids well [50]; limited carotenoid-specific data available.	Promising efficiency and food safety improvements suggest good potential, but carotenoid-specific evidence is still limited.
Greenhouse and tunnel dryers	Enclosed, UV-filtered plastic; airflow and temperature more controlled.	Senegal: 3.8 mg β-carotene/100 g in dried mango; modelling suggests potential for significant dietary contributions [51,52].	High–evidence of strong β-carotene retention and potential for population-level impact if widely adopted.
Solar photovoltaic/electric-assisted dryers (SPE, fan-assisted DF/IF)	Fans/back-up energy stabilize drying; faster drying under variable weather.	Mozambique: fan-assisted dryers dried mango ~40 h faster than open sun, achieved safe aw <0.6; carotenoid retention not yet quantified [39].	Potentially very high–rapid drying likely protects carotenoids; but direct evidence for vitamin A outcomes is lacking.

* Interpretation based on nutrient retention studies. Reported values vary widely, and some very high retention figures likely reflect controlled experimental settings, measurement choices, or concentration effects. Direct links between dryer adoption and reduced VAD prevalence remain to be demonstrated.

## Data Availability

No new data were created or analyzed in this study. Data sharing is not applicable.

## Supplementary Materials

The following supporting information can be downloaded at: https://www.mdpi.com/article/10.3390/foods14223979/s1, Table S1: Quantitative summary of β-carotene or Vitamin A retention (µg/100 g).

## References

[1] World Health Organization (2009). Global Prevalence of Vitamin A Deficiency in Populations at Risk 1995–2005: WHO Global Database on Vitamin A Deficiency. https://iris.who.int/handle/10665/44110.

[2] Stevens G.A., Bennett J.E., Hennocq Q., Lu Y., De-Regil L.M., Rogers L., Danaei G., Li G., White R.A., Flaxman S.R. (2015). Trends and mortality effects of vitamin A deficiency in children in 138 low-income and middle-income countries between 1991 and 2013: A pooled analysis of population-based surveys. Lancet Glob. Health.

[3] Baye K., Laillou A., Seyoum Y., Zvandaziva C., Chimanya K., Nyawo M. (2022). Estimates of child mortality reductions attributed to vitamin A supplementation in sub-Saharan Africa: Scale up, scale back, or refocus?. Am. J. Clin. Nutr..

[4] Zhao T., Liu S., Zhang R., Zhao Z., Yu H., Pu L., Wang L., Han L. (2022). Global burden of vitamin A deficiency in 204 countries and territories from 1990–2019. Nutrients.

[5] Korkalo L., Freese R., Alfthan G., Fidalgo L., Mutanen M. (2015). Poor micronutrient intake and status is a public health problem among adolescent Mozambican girls. Nutr. Res..

[6] Picolo M., Barros I., Joyeux M., Gottwalt A., Possolo E., Sigauque B., Kavle J.A. (2019). Rethinking integrated nutrition-health strategies to address micronutrient deficiencies in children under five in Mozambique. Matern. Child Nutr..

[7] Berde A.S., Bester P., Kruger I.M. (2019). Coverage and factors associated with vitamin A supplementation among children aged 6–59 months in twenty-three sub-Saharan African countries. Public Health Nutr..

[8] Imdad A., Mayo-Wilson E., Haykal M.R., Regan A., Sidhu J., Smith A., Bhutta Z.A. (2022). Vitamin A supplementation for preventing morbidity and mortality in children from six months to five years of age. Cochrane Database Syst. Rev..

[9] Raiten D.J., Darnton-Hill I., Tanumihardjo S.A., Suchdev P.S., Udomkesmalee E., Martinez C., Mazariegos D.I., Mofu M., Kraemer K., Martinez H. (2020). Perspective: Integration to implementation (I-to-I) and the micronutrient forum—Addressing the safety and effectiveness of vitamin A supplementation. Adv. Nutr..

[10] Nonvignon J., Aryeetey G.C., Adjagba A., Asman J., Sharkey A., Hasman A., Pallas S.W., Griffiths U.K. (2023). The political economy of financing traditional vaccines and vitamin A supplements in six African countries. Health Policy Plan..

[11] World Health Organization (2023). Methodology for the Update of the Global Health Expenditure Database 2020–2022. https://apps.who.int/nha/database.

[12] Janmohamed A., Doledec D., Dissieka R., Jalloh U.H., Juneja S., Beye M., Ndiaye F., Jumbe T., Baker M.M. (2024). Vitamin A supplementation coverage and associated factors for children aged 6 to 59 months in integrated and campaign-based delivery systems in four sub-Saharan African countries. BMC Public Health.

[13] Akhtar S., Ahmed A., Randhawa M.A., Atukorala S., Arlappa N., Ismail T., Ali Z. (2013). Prevalence of vitamin A deficiency in South Asia: Causes, outcomes, and possible remedies. J. Health Popul. Nutr..

[14] da Cunha M.D.S.B., Campos Hankins N.A., Arruda S.F. (2019). Effect of vitamin A supplementation on iron status in humans: A systematic review and meta-analysis. Crit. Rev. Food Sci. Nutr..

[15] (2022). Global Nutrition Report: Stronger Commitments for Greater Action. Bristol, UK: Development Initiatives. https://media.globalnutritionreport.org/documents/2022_Global_Nutrition_Report.pdf.

[16] Evans E.A., Ballen F.H., Siddiq M., Siddiq M., Brecht J.K., Sidhu J.S. (2017). Mango production, global trade, consumption trends, and postharvest processing and nutrition. Handbook of Mango Fruit: Production, Postharvest Science, Processing Technology and Nutrition.

[17] USDA (2024). Food Data Central: Mango, Raw. https://fdc.nal.usda.gov..

[18] Akram S., Mushtaq M., Waheed A. (2021). β-Carotene: Beyond provitamin A. A Centum of Valuable Plant Bioactives.

[19] Veda S., Platel K., Srinivasan K. (2007). Varietal differences in the bioaccessibility of β-carotene from mango (*Mangifera indica*) and papaya (*Carica papaya*) fruits. J. Agric. Food Chem..

[20] Varakumar S., Kumar Y.S., Reddy O.V.S. (2011). Carotenoid composition of mango (*Mangifera indica* L.) wine and its antioxidant activity. J. Food Biochem..

[21] Maldonado-Celis M.E., Yahia E.M., Bedoya R., Landázuri P., Loango N., Aguillón J., Restrepo B., Guerrero Ospina J.C. (2019). Chemical Composition of Mango (*Mangifera indica* L.) Fruit: Nutritional and Phytochemical Compounds. Front. Plant Sci..

[22] Kiokias S., Gordon M.H. (2004). Antioxidant properties of carotenoids in vitro and in vivo. Food Rev. Int..

[23] Britton G. (1995). Structure and properties of carotenoids in relation to function. FASEB J..

[24] Schieber A., Carle R. (2005). Occurrence of carotenoid cis-isomers in food: Technological, analytical, and nutritional implications. Trends Food Sci. Technol..

[25] Aparicio-Ruiz R., Mínguez-Mosquera M.I., Gandul-Rojas B. (2011). Thermal degradation kinetics of lutein, β-carotene and β-cryptoxanthin in virgin olive oils. J. Food Compos. Analysis.

[26] Pénicaud C., Achir N., Dhuique-Mayer C., Dornier M., Bohuon P. (2011). Degradation of β-carotene during fruit and vegetable processing or storage: Reaction mechanisms and kinetic aspects: A review. Fruits.

[27] Vásquez-Caicedo A.L., Schilling S., Carle R., Neidhart S. (2007). Effects of thermal processing and fruit matrix on b-carotene stability and enzyme inactivation during transformation of mangoes into purée and nectar. Food Chem..

[28] Palafox-Carlos H., Yahia E., Islas-Osuna M.A., Gutierrez-Martinez P., Robles-Sánchez M., González-Aguilar G.A. (2012). Effect of ripeness stage of mango fruit (*Mangifera indica* L., cv. Ataulfo) on physiological parameters and antioxidant activity. Sci. Hortic..

[29] European Food Safety Authority (2015). Outcome of a public consultation on the Draft Scientific Opinion of the EFSA Panel on Dietetic Products Nutrition Allergies (NDA) on Dietary Reference Values for vitamin A. EFSA Support. Publ..

[30] Codjia G. (2001). Food sources of vitamin A and provitamin A specific to Africa: An FAO perspective. Food Nutr. Bull..

[31] FAO (2011). Global Food Losses and Food Waste–Extent, Causes and Prevention.

[32] Dissa A.O., Bathiebo D.J., Desmorieux H., Coulibaly O., Koulidiati J. (2011). Experimental characterisation and modelling of thin layer direct solar drying of Amelie and Brooks mangoes. Energy.

[33] Malik A.U., Khan A.S., Ahmad I., Singh Z. (2017). Postharvest Handling of Fruits and Vegetables. Horticulture Science and Technology. https://ncfreshproducesafety.ces.ncsu.edu/wp-content/uploads/2014/03/Postharvest-Handling-of-Fruits-and-Vegetables_NCAT.pdf?fwd=no.

[34] Ekechukwu O.V., Norton B. (1999). Review of solar-energy drying systems II: An overview of solar drying technology. Energy Convers. Manag..

[35] Visavale G.L., Hii C.L., Ong S.P., Jangam S.V., Mujumdar A.S. (2012). Principles, classification and selection of solar dryers. Solar Drying: Fundamentals, Applications and Innovations.

[36] Kumar M., Sansaniwal S.K., Khatak P. (2016). Progress in solar dryers for drying various commodities. Renew. Sustain. Energy Rev..

[37] Osunde Z.D. (2017). Effect of pretreatments and drying methods on some qualities of dried mango (*Mangifera indica*) fruit. Agric. Eng. Int. CIGR J..

[38] Nyangena I.O., Owino W.O., Imathiu S., Ambuko J. (2019). Effect of pretreatments prior to drying on antioxidant properties of dried mango slices. Sci. Afr..

[39] Viola Salvador P., Phinney R., Östbring K., Tivana L., Rayner M., Galindo F.G., Davidsson H. (2025). Minimizing post-harvest waste of mango in rural Mozambique—The effect of different solar setups in mango drying. AIMS Agric. Food.

[40] Lebaka V.R., Wee Y.-J., Ye W., Korivi M. (2021). Nutritional Composition and Bioactive Compounds in Three Different Parts of Mango Fruit. Int. J. Environ. Res. Public Health.

[41] Vélez-de la Rocha R., Barajas J.A.S., Cháidez-Quiróz C., Torres F.I.C., Cabanillas E.T., Vergara-Jiménez M.J. (2024). Phytochemicals, antioxidant activity and nutritional profile of pulp, peel and peel fiber of mango (*Mangifera indica* L.) cultivar Ataulfo. Funct. Foods Health Dis..

[42] Lenucci M.S., Tornese R., Mita G., Durante M. (2022). Bioactive Compounds and Antioxidant Activities in Different Fractions of Mango Fruits (*Mangifera indica* L., Cultivar Tommy Atkins and Keitt). Antioxidants.

[43] Khoo H.-E., Prasad K.N., Ismail A., Mohd-Esa N. (2010). Carotenoids from Mangifera Pajang and Their Antioxidant Capacity. Molecules.

[44] Ruales J., Baenas N., Moreno D.A., Stinco M.C., Meléndez-Martínez A.J., García-Ruiz A. (2018). Biological Active Ecuadorian Mango ‘Tommy Atkins’ Ingredients—An Opportunity to Reduce Agrowaste. Nutrients.

[45] Villacís-Chiriboga J., Jacobs G., Camp J.V., Elst K., Ruales J., Marcillo-Parra V., Böhm V., Bunea A., Cirlini M., Craft N. (2022). Interlaboratory exercise for the analysis of carotenoids and related compounds in dried mango fruit (*Mangifera indica* L.). J. Food Compos. Anal..

[46] Mohammed S., Edna M., Siraj K. (2020). The effect of traditional and improved solar drying methods on the sensory quality and nutritional composition of fruits: A case of mangoes and pineapples. Heliyon.

[47] García-Muñoz M.C., Romero-Barrera Y., Amortegui-Sánchez L.F., Villagrán E., Espitia-González J.J., Pedroza-Berrío K.J. (2025). Solar Dehydration of Mangoes as an Alternative for System Sustainability, Food and Nutritional Security, and Energy Transition. Sustainability.

[48] Mongi R.J., Ndabikunze B.K., Wicklund T., Chove L.M., Chove B.E. (2015). Effect of solar drying methods on total phenolic contents and antioxidant activity of commonly consumed fruits and vegetable (mango, banana, pineapple and tomato) in Tanzania. Afr. J. Food Sci..

[49] Mongi R.J. (2023). Physicochemical properties, microbial loads and shelf life prediction of solar dried mango (Mangifera indica) and pineapple (*Ananas comosus*) in Tanzania. J. Agric. Food Res..

[50] Mongi R.J., Ngoma S.J. (2022). Effect of solar drying methods on proximate composition, sugar profile and organic acids of mango varieties in Tanzania. Appl. Food Res..

[51] Rankins J., Sathe S.K., Spicer M.T. (2008). Solar drying of mangoes: Preservation of an important source of vitamin A in French-speaking West Africa. J. Am. Diet. Assoc..

[52] Rankins J., Hopkinson S., Diop M. (1989). Palatability and nutritional significance of solar dried mangoes for Senegal. Ecol. Food Nutr..

[53] Dissa A.O., Bathiebo J., Kam S., Savadogo P.W., Desmorieux H., Koulidiati J. (2009). Modelling and experimental validation of thin layer indirect solar drying of mango slices. Renew. Energy.

[54] Ssemwanga M., Makule E., Kayondo S.I. (2020). Performance analysis of an improved solar dryer integrated with multiple metallic solar concentrators for drying fruits. Sol. Energy.

[55] Akoy E.A.O.M., Ismail M.A., Ahmed E.F.A., Luecke W. Design and Construction of a Solar Dryer for Mango Slices. Proceedings of the International Research on Food Security, Natural Resource Management and Rural Development-Tropentag.

[56] Simate I.N., Cherotich S. (2017). Design and testing of a natural convection solar tunnel dryer for mango. J. Sol. Energy.

[57] Kaméni A., Mbofung C.M., Ngnamtam Z., Doassem J., Hamadou L., Jasmin J.Y., Seiny Boukar L., Floret C. (2003). Aptitude au séchage de quelques variétés de mangue cultivée au Cameroun: Amélie, Zill, Irwin et Horé Wandou. Savanes Africaines: Des Espaces en Mutation, des Acteurs Face à de Nouveaux Défis. Actes du Colloque, Garoua, Cameroun.

[58] Mwamba I., Mputu J.N. (2022). Contribution to the physicochemical and microbiological study of dried mango. Comparison of two drying methods (oven and solar drying). Curr. Overv. Sci. Technol. Res..

[59] Mwamba I., Tshimenga K., Kayolo J., Mulumba L., Gitago G., Tshibad C.M., Noël J., Kanyinda M. (2017). Comparison of two drying methods of mango (oven and solar drying). MOJ Food Process Technol..

[60] Dereje B., Abera S. (2020). Effect of pretreatments and drying methods on the quality of dried mango (*Mangifera indica* L.) slices. Cogent Food Agric..

[61] Mahunu G.K., Appiah F., Abubakari A.H. (2012). Effect of solar and oven drying on the nutrient composition of Keitt mango (*Mangifera indica* L.) pulp. Ghana J. Hortic..

[62] Touré S., Kibangu-Nkembo S. (2004). Comparative study of natural solar drying of cassava, banana and mango. Renew. Energy.

[63] Muoki P.N., Makokha A.O., Onyango C.A., Ojijo N.K. (2009). Potential contribution of mangoes to reduction of vitamin A deficiency in Kenya. Ecol. Food Nutr..

[64] Weber D., Grune T. (2012). The contribution of β-carotene to vitamin A supply of humans. Mol. Nutr. Food Res..

[65] Trumbo P., Yates A.A., Schlicker S., Poos M. (2001). Dietary Reference Intakes. J. Am. Diet. Assoc..

[66] Viola Salvador P., Kugbega S., Lazarte C., Tivana L., Galindo F.G. (2025). Challenges and Drivers for the Adoption of Improved Solar Drying Technologies in Mango Farming: A Case Study of Smallholder Farmers in Mozambique. Sustainability.

[67] Keats E.C., Das J.K., Salam R.A., Lassi Z.S., Imdad A., Black R.E., Bhutta Z.A. (2021). Effective interventions to address maternal and child malnutrition: An update of the evidence. Lancet Child Adolesc. Health.

[68] Low J.W., Arimond M., Osman N., Cunguara B., Zano F., Tschirley D. (2007). A food-based approach introducing Orange-fleshed sweet potatoes increased vitamin A intake and serum retinol concentrations in young children in rural Mozambique. J. Nutr..

[69] Hotz C., Gibson R.S. (2007). Traditional food-processing and preparation practices to enhance the bioavailability of micronutrients in plant-based Diets1. J. Nutr..

[70] Chibuzo N.S., Osinachi U.F., James M.T., Chigozie O.F., Dereje B., Irene C.E. (2021). Technological advancements in the drying of fruits and vegetables: A review. Afr. J. Food Sci..

[71] Bhutta Z.A., Das J.K., Rizvi A., Gaffey M.F., Walker N., Horton S., Webb P., Lartey A., Black R.E. (2013). Evidence-based interventions for improvement of maternal and child nutrition: What can be done and at what cost?. Lancet.

